# Long-term natural course of patients with lymph node station 6 metastasis after pylorus-preserving gastrectomy

**DOI:** 10.1007/s10120-025-01600-2

**Published:** 2025-04-18

**Authors:** Sa-Hong Kim, Franco José Signorini, Kyoyoung Park, Chungyoon Kim, Jeesun Kim, Yo-Seok Cho, Seong-Ho Kong, Do-Joong Park, Hyuk-Joon Lee, Han-Kwang Yang

**Affiliations:** 1 Department of Surgery, Seoul National University Hospital, 101 Daehak-ro, Jongno-gu, Seoul, 03080 Republic of Korea; 2 Private University Hospital of Córdoba, Córdoba, Argentina; 3 Department of Surgery, Seoul National University College of Medicine, Seoul, Republic of Korea; 4 Cancer Research Institute, Seoul National University College of Medicine, Seoul, Republic of Korea

**Keywords:** Pylorus-preserving gastrectomy (PPG), Lymph node station 6 (LN#6), Therapeutic index (TI)

## Abstract

**Background:**

Meticulous lymph node 6 station (LN#6) dissection is mandatory in pylorus-preserving gastrectomy (PPG), but can increase the risk of complications, such as postoperative delayed gastric emptying. With analyzing lymphatic spread patterns based on cross-sectional tumor location, we planned to predict the surgical burden of LN#6 dissection, balancing oncological safety and risk of postoperative complications.

**Methods:**

We included consecutive PPG cases at Seoul National University Hospital (2007–2017) to assess the incidence, 5-year survival rate (5YSR), and 3-year recurrence-free survival (3RFS) of LN#6 metastasis. Cox regression analyzed the impact of LN#6 metastasis itself on 5YSR and 3RFS. The effect of tumor location among gastric middle-third tumors on LN#6 metastasis was evaluated. The therapeutic indices (TI) of LN#6 based on tumor location were calculated.

**Results:**

Among 1070 PPG patients, 5YSR and 3RFS were 97.0% and 98.9%. LN#6 metastasis was found in 11 patients (1.03%), with 3 recurrences observed among them (3/11, 0.28%). LN#6 metastasis itself did not significantly affect 5YSR (*p* = 0.266) or 3RFS (*p* = 0.075). Tumor location showed a significant association for LN#6 metastasis (*p* = 0.015), with low body greater curvature (LB-GC) showing the highest prevalence (5/11, 45.45%). TI of LN#6 for LB-GC tumors was 3.76, while TI for low body lesser curvature (LB-LC) and midbody lesser curvature (MB-LC) tumors was 0.0.

**Conclusions:**

LN#6 metastasis is infrequent and does not affect 5YSR or 3RFS in PPG patients. Tumors in LB-GC demonstrated a higher tendency for lymphatic spread to LN#6, while those in lesser curvature demonstrated a lower spread, suggesting a reduced surgical burden for lesser curvature tumors.

**Mini-abstract:**

This study evaluated LN#6 metastasis in 1070 PPG patients, demonstrating low incidence and favorable oncological outcomes, supporting tailored LN#6 dissection for lesser curvature tumors to minimize complications without compromising safety.

**Supplementary Information:**

The online version contains supplementary material available at 10.1007/s10120-025-01600-2.

## Background

As the extent of lymph node [1] metastasis has been known to be the most significant prognostic factor in gastric cancer, extended lymph node dissection to remove a wider range of lymph nodes for curative intent was adopted. Consequently, D2 lymph node (LN) dissection was widely adopted as the standard treatment for locally advanced gastric cancer [2]. Meantime, nationwide gastric cancer screening programs implemented by Japan and Korea have shown considerable advancements, leading to early diagnosis and better outcomes [3–5]. The 5- and 10-year survival rates for patients with early gastric cancer (EGC) who underwent curative surgery exceed 90% and 85–90%, respectively [6, 7]. In the event of progress in gastric cancer screening program leading to early diagnoses and a consequent reduced incidence of lymph node metastasis, the routine application of D2 LN dissection has been questioned, as it may be an overtreatment for certain patients [8–10].

The excellent outcomes observed in EGC with extended life expectancy led to the advent of function-preserving surgeries, emphasizing both optimal cancer curability and preservation of physiological function [11–14]. Pylorus-preserving gastrectomy (PPG) is one of the function-preserving surgeries and was originally introduced as a surgical procedure for gastric ulcers [11]. PPG has demonstrated functional benefits over standard distal gastrectomy, such as low incidence of gastritis, reflux gastroesophagitis, dumping syndrome, nutritional deficit, weight loss, gallbladder dysfunction, and gallstone sequelae. These benefits arise from the preservation of innervation through the hepatic branch of the vagus nerve and maintenance of blood supply through infrapyloric vessels to the pylorus and antral cuff [12, 15–20]. Eventually, PPG has been recommended as the standard procedure for EGC patients who meet certain criteria [21], with presentation of feasible oncologic outcomes [13, 14, 22–24].

However, PPG was questioned of the potential incomplete LN dissection in the suprapyloric area (lymph node station 5; LN#5) and infrapyloric area (lymph node station 6; LN#6) due to preservation of the pylorus and antral cuff. This incomplete D1 dissection may compromise the oncological safety of gastric cancer surgery and cause consequent poor surgical outcomes. In response to concerns about incomplete lymph node dissection in LN#5 and LN#6, a few studies have been conducted to assess the completeness of these dissections. Notably, a low probability of lymph node metastasis to LN#5 was revealed when the primary tumor was located at the middle third of the stomach [8, 25]. It was eventually allowed to omit LN#5 dissection in PPG, adhering to appropriate indications of PPG [26]. On the other hand, meticulous LN#6 dissection remained mandatory due to somewhat higher LN metastatic rate of LN#6 than that of LN#5 despite adhering to indications of PPG [26]. Nevertheless, concerns about oncologic safety emerge when LN#6 metastasis is identified in postoperative pathology, prompting speculation that additional metastatic lymph nodes could have inadvertently been left behind at LN#6 station in vivo*.* To mitigate this potential risk, meticulous LN#6 dissection is routinely performed. However, this approach can inevitably result in a degree of injury to the infrapyloric vessels, potentially contributing to complications such as delayed gastric emptying—an effect that remains challenging to be quantified.

Tokunaga et al. observed that tumor location tends to align with lymphatic pathways along the corresponding arterial supply, suggesting that patients indicated for PPG who have tumors located further from the greater curvature might exhibit a lower likelihood of LN#6 metastasis. In other words, if LN#6 metastasis follows a favorable natural course, it may be feasible to predict and tailor the surgical burden of LN#6 dissection based on tumor location.

Therefore, we aimed to demonstrate the 5-year survival rate (5YSR) and recurrence-free survival rate (RFS) of PPG patients. We calculated the incidence of LN#6 metastasis and assessed the impact of LN#6 metastasis itself on 5YSR and 3RFS. Additionally, we investigated the association between tumor locations and LN#6 metastasis and calculated the therapeutic index, to predict the surgical burden of LN#6 dissection during PPG.

## Methods

### Patients

We conducted a retrospective review of the electronic medical records (EMRs) for 1070 patients who met the indication for pylorus-preserving gastrectomy (PPG), clinically staged as T1N0M0, and underwent PPG for pathologically proven gastric adenocarcinoma at Seoul National University Hospital between 2007 and 2017.

Baseline demographic variables, including age, sex, the number of retrieved and metastatic lymph nodes, follow-up period, surgical approach, clinical stage, gross type of the tumor, tumor location (longitudinal and cross-sectional), pathological characteristics (differentiation, Lauren classification, lymphatic invasion, venous invasion, perineural invasion), and TNM stage were reviewed.

### Procedures for pylorus-preserving gastrectomy

The procedure starts with omentectomy, followed by ligation and lymph node retrieval along the left gastroepiploic vessels (LN#4sb) and the right gastroepiploic vessels (LN#4d). At the root of the right gastroepiploic vessels (infrapyloric area; LN#6), careful dissection is mandatory to preserve the infrapyrloic vessels. The ligation point of the right gastroepiploic artery depends on the origin of infrapyloric arteries (proximal, distal, and caudal type) [27, 28]. The suprapyrloic area (LN#5) should be handled cautiously to avoid injuries to the pyloric and hepatic branches from the vagus nerve and right gastric vessels. The right gastric vessels are ligated after branching off the first or second arcade. The LN#7, #8a, #9, and optionally #11p are dissected with ligation of the coronary vein and left gastric artery. The right paracardial LNs (LN#1) and lesser curvature LNs (LN#3a and 3b) are dissected with high caution to preserve the innervating nerves and supplying vessels to the gastric pylorus. Stomach transection ensures 3 cm of antral cuff with an additional 2 cm of oncological safety margin, followed by end-to-end gastrogastrostomy.

### Oncological outcomes

In terms of oncological outcomes, the 5-year survival rate (5YSR) and 3-year recurrence-free survival (3RFS) were calculated from the date of surgery. Patients who did not visit the outpatient department within 5 years of achieving no evidence of disease (NED) were considered “lost to follow-up.” Recurrence date was defined as the date when a certain recurrence was identified with any diagnostic modality, such as ultrasonography, computed tomography (CT), positron emission tomography (PET), or esophagogastroduodenoscopy.

### Incidence and impact of lymph node metastasis of each topological lymph node station on survival and recurrence

The incidence for lymph node (LN) metastasis to each station was calculated. Additionally, we assessed the impact of LN metastasis of each LN station itself on 5YSR and 3RFS. We evaluated the D1 + stations of PPG, including LN#1, #3a, #3b, #4sb, #4d, #6, #7, #8a, and #9, and optionally #11p.

### Tumor location of the gastric middle-third tumors and LN#6 metastasis

Among the patients with gastric middle-third tumors, we categorized the patients into eight groups according to the longitudinal tumor location (midbody; MB and low body; LB) and circumferential tumor location (lesser curvature; LC, greater curvature; GC, anterior wall; AW, and posterior wall; PW). We assessed the association between tumor location and LN metastasis to each topological LN station.

The lymphatics from the tumors tend to follow the corresponding lymphatic draining routes associated with supplying vessels [29]. We calculated the therapeutic index (TI), a tool for quantifying the necessity of dissecting a certain lymph node station based on the primary tumor location (Supplementary Fig. 1). We calculated the TI of LN#6 station based on the aforementioned eight groups, and additionally based on four groups (midbody and low body by lesser curvature and the others), and two groups (all lesser curvature and all the others) [8].

### Statistical analysis

Descriptive statistics were used to evaluate the baseline characteristics of the patients. 5YSR and 3RFS were evaluated using Kaplan–Meier method. The distribution of tumor locations, both longitudinal and cross-sectional, was analyzed with Chi-square test. Univariate and multivariate analyses were performed to find out the impact of LN metastasis of each station itself on oncologic outcomes (5YSR and 3RFS) using Cox proportional hazards model. The association between tumor locations among gastric middle-third tumors and LN metastasis of each station was evaluated using Chi-square test for independence with multiple categorical variables. *p* value less than 0.05 (*p* < 0.05) was considered statistically significant. Analyses and graphical illustrations were conducted using SPSS software version 21.0 (IBM Corp., Armonk, NY, USA) and R Studio (R Foundation for Statistical Computing, Vienna, Austria).

### Ethical approval

The data collection and analysis of this study were approved by the Institutional Review Board (IRB) of the Seoul National University Hospital (IRB No.: H-1905-042-103).

## Results

### Baseline demographics

The data of 1,070 patients who underwent PPG were reviewed. The mean age of the PPG patients was 56.95 ± 11.98. There was 539 male patients (50.4%) and 531 female patients (49.6%). The average number of resected and metastatic LNs were 36.76 ± 12.50 and 0.33 ± 1.72, respectively (Table 1).

**Table 1:** Baseline characteristics of 1,070 patients who underwent PPG

Characteristic	Number	Percentage
Age (year ± SD)	56.95 ± 11.98	
Sex		
Male/female	539/531	50.4/49.6
Retrieved LN (mean ± SD)	36.76 ± 12.50	
Metastatic LN (mean ± SD)	0.33 ± 1.72	
Follow-up period (mean ± SD)	7.31 ± 2.45	
Minimum	0.02	
Maximum	13.98	
Approach		
Open	85	7.9
Laparoscopic	825	77.1
Robotic	159	14.9
Open conversion	1	0.1
Clinical stage^a^		
cT1aN0	319	29.8
cT1bN0	684	63.9
cT1N0	61	5.7
cT2N0	6	1.6
Location (longitudinal)		
High body	65	6.1
Midbody	309	28.9
Low body	488	45.6
Angle	138	12.9
Antrum	70	6.5
Location (cross-sectional)		
Lesser curvature	277	25.9
Greater curvature	253	23.6
Anterior wall	224	20.9
Posterior wall	316	29.5
Gross type^b^		
EGC I	12	1.1
EGC IIa	43	4.0
EGC IIb	194	18.1
EGC IIc	652	60.9
EGC III	7	0.7
EGC, combined gross type	92	8.6
Borrmann I	13	1.2
Borrmann II	7	0.7
Borrmann III	29	2.7
Borrmann IV	2	0.2
Others	19	1.8
Differentiation		
Differentiated	314	29.3
Undifferentiated	594	55.6
Others	162	15.1
Lauren classification		
Intestinal	380	35.5
Diffuse	581	54.3
Mixed	92	8.6
Others	17	1.6
Lymphatic invasion		
No	957	89.4
Yes	110	10.3
Venous invasion		
No	1,056	98.7
Yes	11	1.0
Perineural invasion		
No	1,019	95.2
Yes	48	4.5
T stage		
T1a	585	54.5
T1b	398	37.1
T2	67	6.2
T3	16	1.5
T4a	4	0.4
T4b	0	0
N stage		
N0	981	91.7
N1	48	4.5
N2	21	2.0
N3a	17	1.6
N3b	3	0.3
TNM stage (AJCC 8th)		
IA	921	86.1
IB	84	7.9
IIA	29	2.7
IIB	26	2.4
IIIA	5	0.5
IIIB	5	0.5
IIIC	0	0.0

*SD* standard deviation; *EGC* early gastric cancer

^a^Patients classified as cT1N0 were determined based on CT reports, as they underwent surgery without endoscopic ultrasonography (EUS) or had already undergone endoscopic submucosal dissection (ESD), making EUS evaluation infeasible. All six patients with cT2N0 were determined based on EUS findings, but their CT findings exhibited cT1 or lesser than cT1.

^b^ The gross type of the specimen was documented in the pathologic report. If the pathologic examination confirmed advanced gastric cancer (AGC), the pathologists accordingly reported the lesion based on Borrmann type I, II, III, or IV. Nineteen patients were reported as undetermined gross type due to prior ESD and were categorized as 'Others'

The average follow-up period of the study was 7.31 ± 2.45 years. During the follow-up, recurrence was observed in 24 patients. Among these cases, 17 patients had no metastatic LN, while the other 7 patients were confirmed to have metastatic LNs in their pathologic reports (not shown in the table).

The clinical stages were distributed as cT1aN0 in 29.8% (*n* = 319), cT1bN0 in 63.9% (*n* = 684), cT1N0 in 5.7% (*n* = 61), and cT2N0 in 1.6% (*n* = 6). Patients classified as cT1N0 were determined based on CT reports, as they underwent surgery without endoscopic ultrasonography (EUS) or had already undergone endoscopic submucosal dissection (ESD), making EUS evaluation infeasible. All cT2N0 patients were classified based on EUS findings, but their CT findings exhibited cT1N0 or lesser than cT1. Regarding the longitudinal tumor location, the distribution was significantly uneven (*p* < 0.001, not shown in the table), with over 70% of the tumors situated in the middle-third of the stomach, including midbody (*n* = 309, 28.9%) and low body (*n* = 488, 45.6%). The distribution of tumors based on cross-sectional locations was also significantly uneven (*p* < 0.001, not shown in the table). Specifically, tumors were located on the lesser curvature (LC) in 25.9% (*n* = 277) of cases, on the greater curvature (GC) in 23.6% (*n* = 253), on the anterior wall in 20.9% (*n* = 224), and on the posterior wall in 29.5% (*n* = 316).

The gross type of the specimen was documented in the pathologic report. For patients initially meeting PPG indications through preoperative evaluation, but later confirmed as advanced gastric cancer (AGC) upon pathologic examinations, the lesions were classified by pathologists according to Borrmann type (type I: *n* = 13, type II: *n* = 7, type III: *n* = 29, type IV: *n* = 2). Patients had T1a cancer in 54.5% (*n* = 585) of cases and T1b cancer in 37.1% (*n* = 398) of cases. 91.7% (*n* = 981) of patients showed no evidence of nodal metastasis (N0) postoperatively. Regarding the TNM staging, 86.1% of the patients (*n* = 921) were classified as stage IA and 7.9% (*n* = 84) as stage IB, indicating that a cumulative total of 94.0% were diagnosed with stage I cancer. Further details of the other clinicopathologic data are given in Table 1.

### Oncological outcomes

**Fig.1:**
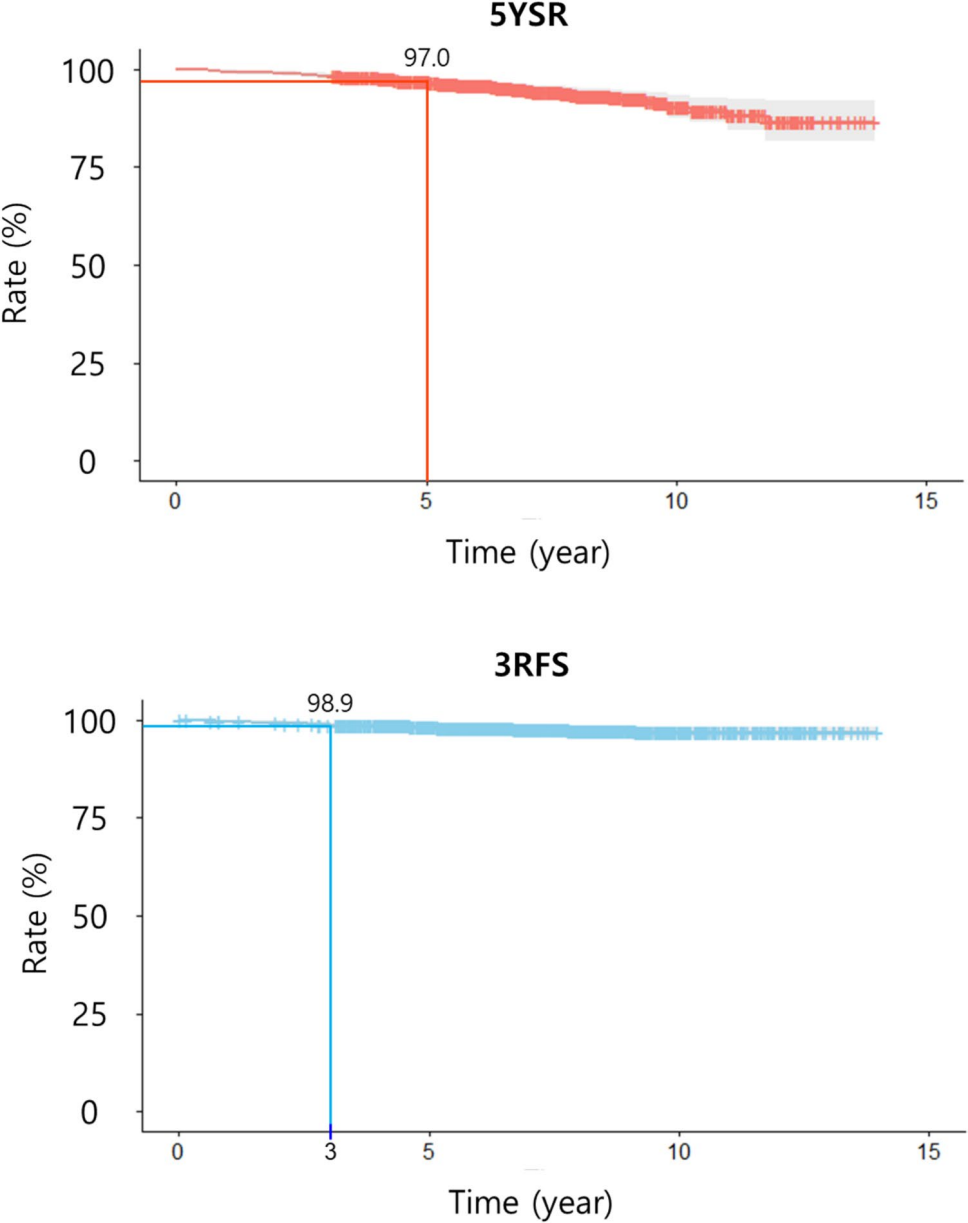
5-year survival rate (5YSR) and 3-year recurrence-free survival (3RFS) in PPG patients

The 5-year survival rate and 3-year recurrence-free survival rate of total PPG patients were 97.0% and 98.9%, respectively (Fig. 1).

### Incidence and impact of lymph node metastasis of each topological lymph node station on survival and recurrence

The incidence of LN metastasis at each topological lymph node station, as described in Table 2, shows that LN#6 metastasis occurred in 1.03% (11 out of 1070 patients). Patient distribution based on tumor locations and topological lymph node station is described in Supplementary Table S1. The incidence of LN metastasis among D1 + LN stations was significantly different using the Chi-square method (*p* < 0.001), and the contribution of each LN station to the Chi-square value is detailed in Supplementary Table S2, where it is noted that LN#6 metastasis accounted for 3.44% of the total contribution.

**Table 2:** Impact of lymph node metastasis of each station on survival and recurrence

LN station	Number of patients dissected/total	Number of patients LN Involved	Univariate (survival)	Multivariate (survival)	Univariate (recurrence)	Multivariate (recurrence)
LN#1	1,062/1,070	24 (2.26)	0.005	0.029	0.012	0.110
LN#3	1,061/1,070	38 (3.58)	0.003	0.048	0.007	0.048
LN#4sb	1,039/1,070	2 (0.19)	0.675		0.061	
LN#4d	1,058/1,070	38 (3.59)	0.639		0.016	0.056
LN#6	1,070/1,070	11 (1.03)	0.005	0.266	0.048	0.075
LN#7	1,062/1,070	24 (2.26)	0.014	0.220	0.025	0.088
LN#8a (D1 +)	1,018/1,070	8 (0.79)	0.014	0.327	0.106	
LN#9 (D1 +)	1,023/1,070	8 (0.78)	0.218		0.052	
LN#11p	844/1,070	4 (0.37)	0.053		0.008	< 0.001

Univariate analysis showed that LN metastasis in LN#1, LN#3, LN#6, LN#7, and LN#8a significantly impacted 5YSR (*p* < 0.05), but multivariate analysis identified only LN#1 and LN#3 as significant factors (*p* = 0.029 and *p* = 0.048, respectively) (Table 2). For 3RFS, univariate analysis revealed significant impacts of LN#1, LN#3, LN#4d, LN#6, LN#7, and LN#11p (*p* < 0.05), while multivariate analysis identified only LN#3 and LN#11p as significant factors (*p* = 0.048 and *p* < 0.001, respectively) (Table 2).

### Tumor location of gastric middle-third tumors and LN#6 metastasis

Analysis of the association between tumor locations and LN metastasis of each topological LN station showed statistical significance only for LN#6 (*p* = 0.015) (Table 3). Tumors at LB-GC showed a higher observed number of LN metastasis than expected (Table 3, Supplementary Table S1).

**Table 3:** Association between tumor locations and LN metastasis of each topological LN station

**LN station**	***p*** **value**^**a**^
LN#1	0.993
LN#3	1.000
LN#4sb	0.260
LN#4d	0.151
LN#6	0.015
LN#7	0.998
LN#8a	0.522
LN#9	0.946
LN#11p	0.161

	**LN#6 metastasis (expected)**^b^	**LN#6 metastasis (observed)**^**b**^
MB-LC	0.93	0
MB-GC	0.64	0
MB-AW	0.51	0
MB-PW	1.10	0
LB-LC	1.16	0
LB-GC	1.37	5
LB-AW	1.17	2
LB-PW	1.32	1

*HB* high body; *MB* midbody; *LB* low body; *LC* lesser curvature; *GC* greater curvature; *AW* anterior wall; *PW* posterior wall

A Chi-square test of independence, a method of assessing relationships between multiple categorical variables, was conducted

^a^Impact of tumor location of gastric middle-third tumors (MB-LC, MB-GC, MB-AW, MB-PW, LB-LC, LB-GC, LB-AW, and LB-PW) on LN metastasis of each topological LN station

^b^The expected and observed frequencies of LN#6 metastasis based on tumor locations, derived from the Chi-square test of independence with multiple categorical variables

Table 4 provides more detailed information of these 11 patients with LN#6 metastasis. Three patients (0.28%, 3 out of 1070) experienced recurrence, 2 of them had tumors on LB-GC, and the other on LB-PW. These three patients were presumed to have advanced disease at the time of the surgery, referring to their extensive LN metastasis. The mean follow-up period was 7.30 ± 1.14 years for these three patients, with a mean overall recurrence-free survival of over 5 years (5.29 ± 0.91). All 11 patients with LN#6 metastasis did not undergo additional completion surgery irrespective of their final stage.

**Table 4:**
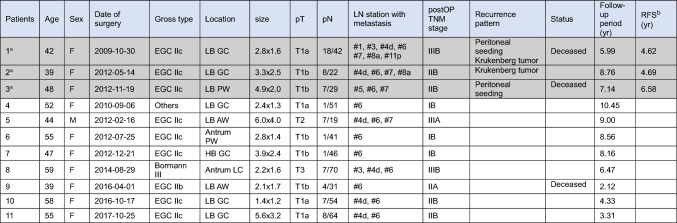
Detailed information of the 11 patients with LN#6 metastasis

*HB* high body; *MB* midbody; *LB* low body; *LC* lesser curvature; *GC* greater curvature; *AW* anterior wall; *PW* posterior wall; *RFS* recurrence-free survival

^a^ Three patients who experienced recurrence are highlighted in gray color

^b^ The mean recurrence-free survival of patients with recurrence was over 5 years (5.29 ± 0.91)

### Therapeutic index

The therapeutic indices (TI) of LN#6 for gastric middle-third tumors were calculated for eight groups, four groups, and two groups. In the eight groups, the TI of LN#6 for low body greater curvature (LB-GC) tumors was 3.76, indicating a higher value compared to the TI of LN#6 for all PPG patients (TI = 1.00). The TI of LN#6 for midbody greater curvature (MB-GC) was 0.00. The TIs of LN#6 for both low body lesser curvature (LB-LC) and midbody lesser curvature (MB-LC) were 0.0. TIs of LN#6 for low body anterior wall (LB-AW), midbody anterior wall (MB-AW), low body posterior wall (LB-PW), and midbody posterior wall (MB-PW) were 0.88, 0.00, 0.78, and 0.00, respectively (Table 5a). TI of LN#6 for the other low body tumors except for lesser curvature was 1.86 (Table 5b), and TI of LN#6 for all tumors except for lesser curvature was 1.18 (Table 5c).

**Table 5:** Therapeutic index of LN#6 for gastric middle-third tumors

**Table 5a**		**Frequency of LN#6 metastasis (%)**	**5YSR of LN#6 metastasis (%)**	**Therapeutic index**
**Category**				
All PPG patients		11/1,070 (1.03)	90.9	1.00
Low body	Greater curvature	5/133 (3.76)	100.0	3.76
Midbody	Greater curvature	0/62 (0.00)	100.0	0.00
Low body	Lesser curvature	0/113 (0.00)	100.0	0.00
Midbody	Lesser curvature	0/90 (0.00)	100.0	0.00
Low body	Anterior wall	2/114 (1.75)	50.0	0.88
Midbody	Anterior wall	0/50 (0.00)	100.0	0.00
Low body	Posterior wall	1/128 (0.78)	100.0	0.78
Midbody	Posterior wall	0/107 (0.00)	100.0	0.00

**Table 5b**		**Frequency of LN#6 metastasis (%)**	**5YSR of LN#6 metastasis (%)**	**Therapeutic index**
**Category**				
All PPG patients		11/1,070 (1.03)	90.9	1.00
Low body	Lesser curvature	0/113 (0.00)	100.0	0.00
Midbody	Lesser curvature	0/90 (0.00)	100.0	0.00
Low body	The others	8/375 (2.13)	87.5	1.86
Midbody	The others	0/219 (0.00)	100.0	0.00

**Table 5c**		**Frequency of LN#6 metastasis (%)**	**5YSR of LN#6 metastasis (%)**	**Therapeutic index**
**Category**				
All PPG patients		11/1,070 (1.03)	90.9	1.00
All	Lesser curvature	0/203 (0.00)	100.0	0.00
All	The others	8/594 (1.35)	87.5	1.18

## Discussion

In pylorus-preserving gastrectomy (PPG), it is essential to select appropriate candidates according to guidelines through preoperative evaluation, and endoscopic ultrasonography (EUS) serves a pivotal role in this assessment. As EUS is capable of differentiating the penetration depth of the gastrointestinal cancers and visualizing the presence of LN involvement or adjacent organ infiltration, it is widely used in practice for preoperative evaluation of gastric cancer [30, 31]. The diagnostic accuracy of T and N staging examined by EUS varies widely (42.6–92% and 63–85%, respectively) [1, 32–35]. However, in our data, patients who met PPG indications based on preoperative evaluation were confirmed to be 91.6% T1 (T1a: 54.5%, *n* = 585; T1b: 37.1%, *n* = 398). Additionally, 91.7% (*n* = 981) of patients showed no evidence of nodal metastasis (N0), and 94% had stage I gastric cancer (stage IA: 86.1%, *n* = 921; stage IB: 7.9%, *n* = 84). These results represented that the selection of PPG candidates is appropriate in our practice based on the PPG indications, resulting in minimal preoperative understaging.

Our data exhibited favorable 5YSR (97.0%) and 3RFS (98.9%) (Fig. 1) compared to previous studies [36–39]. Our data demonstrated that the incidence of LN#6 metastasis after PPG was considerably low (1.03%) (Table 2). Although a significant difference in metastasis rates among D1 + LN stations was observed (*p* < 0.001), the contribution of LN#6 metastasis to the overall Chi-square statistic was minimal, accounting for only 3.44% (Supplementary Table S2). Furthermore, the impact of LN#6 metastasis itself on both 5YSR and 3RFS was not statistically significant according to multivariate analysis (Table 2). Additionally, patients with LN#6 metastasis, whose final stage was revealed to be equal to or higher than stage II, received only adjuvant chemotherapy without additional completion surgery, and eventually achieved a long-term follow-up period. These findings suggest that even if LN#6 metastasis is detected in the postoperative pathology report after PPG, patients can still be monitored with an expectation of a favorable oncological prognosis without deterioration of 5YSR and 3RFS. Nevertheless, we should implement more cautious surveillance strategy to the patients whose LN metastasis patterns were already extensive at the time of surgery for potential recurrence (Table 4).

We divided the patients into eight groups according to longitudinal tumor location (midbody and low body) and circumferential tumor location (lesser curvature, greater curvature, anterior wall, and posterior wall) and analyzed the association between tumor locations and LN metastasis. A significant association between tumor location and LN#6 metastasis was demonstrated, and LB-GC tumors tended to exhibit higher frequency of LN#6 metastasis compared to the other tumor locations (Table 3).

Tokunaga et al. suggested a correlation between tumor location and lymphatic stream along the corresponding supply artery, noting that LN#5 metastasis tended to have their tumors near the lesser curvature (LC) [29]. Following this concept, we subdivide the patients based on cross-sectional tumor location to quantify the association between LN#6 metastasis and LB-GC using the therapeutic index (TI), which has been a crucial concept in justifying the omission of LN#5 dissection in PPG [8, 26]. Notably, the TI of LN#6 for LB-GC tumors was 3.76, substantially higher than TI for all PPG patients, which was 1.00. This high TI for LB-GC tumors underscores the importance of meticulous LN dissection around the right gastroepiploic artery for tumors on the GC side. On the other hand, the TIs of LN#6 for low body lesser curvature (LB-LC) and midbody lesser curvature (MB-LC) tumors were both 0.0, suggesting a lower likelihood of lymphatic spread to LN#6 in lesser curvature tumors (Table 5a). When the four-group categorization was applied, a similar trend was observed. Low body tumors except for lesser curvature showed a higher TI (1.86) compared to low body lesser curvature, although the value was slightly lower than that of LB-GC tumors (Table 5a). Similarly, when comparing all lesser curvature tumors to all the other tumors, the trend remained consistent, with the TI for all the other tumors reported as 1.18. However, the inclusion of midbody tumors, where LN#6 metastasis was not observed, may have diluted the overall trend (Table 5c). Based on the findings from Tables 4 and 5, patients with LB-GC tumors demonstrate a noticeable trend of LN#6 metastasis, suggesting that LN#6 dissection should not be overlooked in these patients.

During PPG, the attempt to preserve the infrapyloric vessels while performing meticulous LN#6 dissection may cause inevitable thermal injury, which cannot be quantitatively assessed, potentially leading to complications such as delayed gastric emptying. However, given the notably low incidence of LN#6 metastasis, along with evidence that its impact on oncological outcomes is not significantly compromised, the fact that low body greater curvature tumors are more likely to demonstrate LN#6 involvement while lesser curvature tumors have minimal likelihood of lymphatic spread to LN#6 station suggests the reduced surgical burden of LN#6 dissection, particularly in cases of lesser curvature tumors. This approach enables a tailored strategy that balances oncological safety with minimizing postoperative complications, such as delayed gastric emptying. Furthermore, similar to how LN#5 dissection was previously omitted in PPG based on the concept of the therapeutic index, these findings provide new insights into LN#6 dissection during PPG, especially when the tumor is located in the lesser curvature.

This study has a few limitations. First, as this study is a retrospective analysis of 1070 patients who already underwent PPG with routine LN#6 dissection, providing direct comparisons of complications, including delayed gastric emptying, based on whether LN#6 dissection was performed was inherently limited. Further studies, such as RCTs, are needed. Second, the limited number of patients with LN metastasis at each topological station, including only 11 patients with LN#6 metastasis, posed challenges in achieving sufficient statistical power. While this study may raise the concept of omitting LN#6 dissection for lesser curvature tumors, the small sample size limits its explanatory power. Therefore, with further studies involving larger sample sizes and more LN#6 metastasis cases, the feasibility of omitting LN#6 dissection could be further evaluated. Third, the lower incidence of metastasis in LN#8, LN#9, and LN#11p compared to LN#6 raises the possibility of omitting dissection of these stations, suggesting that the extent of lymph node dissection could be reduced from D1 + to D1 in PPG-indicated patients, whose clinical stage was T1N0M0. As the incidence of those LN metastasis is low, further studies with larger sample size is necessary. Lastly, while the therapeutic index (TI) offers a useful framework for evaluating the necessity of LN dissection, its application in early gastric cancer requires cautious interpretation. Since TI is a simple combination of LN metastasis rates and 5YSR, and even a single mortality event at a certain LN station can cause a significant drop in 5YSR because of low incidence of LN metastasis at each station, the explanatory power of the TI can be limited.

The novelty of this study is that the evaluations were based on long-term and well-organized data of the natural courses of 1070 PPG patients. To the best of our knowledge, this is the first study conducted in a cohort of over 1000 PPG patients and the first to analyze the therapeutic indices of LN stations in PPG patients based on cross-sectional tumor location. These findings can provide valuable insights for surgeons to tailor the surgical burden of LN#6 dissection according to tumor location, particularly for lesser curvature tumors.

## Supplementary Information

Below is the link to the electronic supplementary material.

Supplementary file 1 (DOCX 13 KB)

Supplementary file 2 (TIF 32 KB)

Supplementary file 3 (DOCX 22 KB)
