# Supplementary material for: Long-term natural course of patients with lymph node station 6 metastasis after pylorus-preserving gastrectomy
Source: Gastric Cancer. 2025 Apr 18;28(4):673–83. doi: 10.1007/s10120-025-01600-2 (PMC12174248; doi:10.1007/s10120-025-01600-2)
Supplement: Supplementary file 1 — (DOCX 13 KB) [file 10120_2025_1600_MOESM1_ESM.docx]

**Supplementary Figure Legends**

**Supplementary Fig. 1** The formula of therapeutic index (TI)
