# Supplementary material for: Long-term natural course of patients with lymph node station 6 metastasis after pylorus-preserving gastrectomy
Source: Gastric Cancer. 2025 Apr 18;28(4):673–83. doi: 10.1007/s10120-025-01600-2 (PMC12174248; doi:10.1007/s10120-025-01600-2)
Supplement: Supplementary file 3 — (DOCX 22 KB) [file 10120_2025_1600_MOESM3_ESM.docx]

**Supplementary Table S1** Incidence of lymph node metastasis based on topological lymph node station; patient distribution based on tumor locations and lymph node station of metastasis

| **Location** | Patient number according to tumor location (%) | Number of patients with LN metastasis to each D1+ stations (%) | | | | | | | | |
| --- | --- | --- | --- | --- | --- | --- | --- | --- | --- | --- |
|  |  | LN#1 | LN#3 | LN#4sb | LN#4d | LN#6 | LN#7 | LN#8a | LN#9 | LN#11p |
| HB-LC | 6 (0.56) | 0 | 0 | 0 | 0 | 0 | 0 | 0 | 0 | 0 |
| HB-GC | 18 (1.68) | 1 | 0 | 0 | 0 | 1 | 1 | 0 | 0 | 0 |
| HB-AW | 4 (0.37) | 0 | 0 | 0 | 0 | 0 | 0 | 0 | 0 | 0 |
| HB-PW | 37 (3.45) | 1 | 2 | 1 | 1 | 0 | 2 | 1 | 1 | 1 |
| MB-LC* | 90 (8.41) | 2 | 5 | 1 | 0 | 0 | 5 | 0 | 1 | 0 |
| MB-GC* | 62 (5.79) | 2 | 2 | 0 | 3 | 0 | 1 | 0 | 1 | 0 |
| MB-AW* | 50 (4.67) | 4 | 3 | 0 | 2 | 0 | 0 | 0 | 0 | 0 |
| MB-PW* | 107 (10.00) | 2 | 2 | 0 | 1 | 0 | 2 | 0 | 2 | 0 |
| LB-LC* | 113 (10.56) | 2 | 4 | 0 | 1 | 0 | 1 | 0 | 0 | 0 |
| LB-GC* | 133 (12.43)^†^ | 2 | 3 | 0 | 14 | 5 | 4 | 3 | 1 | 2 |
| LB-AW* | 114 (10.65) | 2 | 5 | 0 | 8 | 2 | 3 | 0 | 2 | 0 |
| LB-PW* | 128 (11.96) | 2 | 7 | 0 | 3 | 1 | 4 | 1 | 0 | 0 |
| Antrum-LC | 9 (0.84) | 0 | 1 | 0 | 1 | 1 | 0 | 0 | 0 | 0 |
| Antrum-GC | 39 (3.64) | 0 | 0 | 0 | 3 | 0 | 0 | 0 | 0 | 0 |
| Antrum-AW | 13 (1.21) | 0 | 1 | 0 | 0 | 0 | 0 | 0 | 0 | 0 |
| Antrum-PW | 9 (0.84) | 0 | 0 | 0 | 0 | 1 | 0 | 0 | 0 | 0 |
| Angle-LC | 59 (5.51) | 2 | 1 | 0 | 0 | 0 | 0 | 0 | 0 | 0 |
| Angle-GC | 1 (0.09) | 0 | 0 | 0 | 0 | 0 | 0 | 0 | 0 | 0 |
| Angle-AW | 42 (3.93) | 0 | 1 | 0 | 1 | 0 | 1 | 1 | 0 | 1 |
| Angle-PW | 36 (3.36) | 2 | 1 | 0 | 0 | 0 | 0 | 2 | 0 | 0 |
| Total | 1,070 (100.0) | 24 (2.26) | 38 (3.58) | 2 (0.19) | 38 (3.59) | 11 (1.03) | 24 (2.26) | 8 (0.79) | 8 (0.78) | 4 (0.37) |

HB, high body; MB, midbody; LB, low body; LC, lesser curvature; GC, greater curvature; AW, anterior wall; PW, posterior wall

A total of 1,070 patients are presented in a cross-table format, with rows representing the tumor location as pairs of longitudinal and cross-sectional locations, and columns representing the topological lymph node stations. The orange-colored zone indicates the incidence of LN metastasis to each station.

The difference of LN metastasis rates among the stations was *p*<0.001 with Chi-square test.

* The number of the patients with the gastric middle-third tumor was 797, comprising 74.49% of the total number of patients (797 out of 1,070 cases).

^†^ The most common location among the tumors at middle-third of the stomach was the greater curvature side of the low body (LB-GC), with 133 out of 797 cases (16.69%).

**Supplementary Table S2** The contribution of each LN station to the overall Chi-square statistic

| **LN metastasis** | **Value (%)** |
| --- | --- |
| #1 | 2.37 |
| #3 | 26.77 |
| #4sb | 16.82 |
| #4d | 26.99 |
| #6 | 3.44 |
| #7 | 2.37 |
| #8a | 6.06 |
| #9 | 6.14 |
| #11p | 9.04 |

The contribution of LN#6 metastasis to the overall Chi-square statistic was 3.44%.
